# Can we do better at measuring patient-reported outcomes after cranioplasty? A systematic review

**DOI:** 10.1007/s10143-023-02006-3

**Published:** 2023-05-06

**Authors:** Taborah Z. Zaramo, Ian Zelko, Dashaun Ragland, Jude Tunyi, Manraj N. Kaur, Nojan Bajestani, Clara N. Lee, Kevin C. Chung, Kerry-Ann S. Mitchell

**Affiliations:** 1 Department of Plastic & Reconstructive Surgery, The Ohio State University Wexner College of Medicine, Columbus, OH USA; 2 Department of Plastic Surgery, Cleveland Clinic, Cleveland, OH USA; 3 Department of Surgery, Brigham and Women’s Hospital, Harvard Medical School, Boston, MA USA; 4 Section of Plastic Surgery, Department of Surgery, University of Michigan, Ann Arbor, MI USA

**Keywords:** Cranioplasty, Quality of life, Patient-reported outcome measure, Decompressive craniectomy, Neuroplastic surgery, Health-related quality of life, Traumatic brain injury, Stroke

## Abstract

Measuring quality of life (QOL) after cranioplasty is increasingly evident as a necessary component of patient-centered care. For data to be useful in clinical decision-making and approval of new therapies, studies must utilize valid and reliable instruments. Our objective was to critically appraise studies evaluating QOL in adult cranioplasty patients and determine validity and relevance of the patient-reported outcome measures (PROMs) used. Electronic databases of PubMed, Embase, CINAHL, and PsychINFO were used to identify PROMs measuring QOL in adult patients with cranioplasty. The methodological approach, cranioplasty outcomes, and domains measured by the PROMs were extracted and summarized descriptively. A content analysis of the identified PROMs was completed to identify the concepts measured. From 2236 articles identified, 17 articles containing eight QOL PROMs met the inclusion criteria. None of the PROMs was specifically validated or developed for adults undergoing cranioplasty. The QOL domains included physical health, psychological health, social health, and general QOL. These four domains encompassed 216 total items among the PROMs. Appearance was only assessed in two PROMs. To our knowledge, there are currently no validated PROMs that comprehensively measure appearance, facial function, and adverse effects in adults undergoing cranioplasty. There is an urgent need to develop PROMs to measure QOL outcomes rigorously and comprehensively in this patient population to inform clinical care, research, and quality improvement initiatives. Findings from this systematic review will be used to derive an outcome instrument containing important concepts related to QOL in patients who undergo cranioplasty.

## Introduction

Cranioplasty is a common procedure to repair a skull defect resulting from a previous operation or injury [1, 2]. While the procedure is primarily performed to protect the brain from outside forces, cranioplasty can improve neurological function, enhance glymphatic fluid circulation, and restore intracranial pressure adaptations and cerebrospinal fluid circulation [3, 4]. Recent studies have also highlighted the impact of cranioplasty on syndrome of the trephined or “sinking flap syndrome.” This syndrome refers to the neurological deterioration that occurs after a large craniectomy, with various symptoms including headaches, motor weakness, worsened hemisyndrome, language deficits, and cognitive disorders with or without an orthostatic component. These symptoms can improve or resolve entirely as early as 3–4 days after a cranioplasty [1, 2, 5–7].

Although the neuroprotective functions of cranioplasty are critically important, cranioplasty is also an important rehabilitation procedure that can improve a patient’s quality of life by restoring the appearance of the skull. An abnormal calvarial shape can affect how patients view themselves and how they are perceived by others. Such perceptions have important implications for psychological well-being, distress, and social performance [8].

Despite the necessity and frequency of cranioplasty, it has substantial risk. A national analysis database study of 8275 patients reported that more than one-third of individuals who underwent cranioplasty experienced perioperative complications [1]. Older age, larger cranioplasty size, and delayed timing were associated with higher complication risk overall. Twenty-six percent of complications, including wound dehiscence, wound infection, implant exposure, and bleeding related complications were specifically related to the cranioplasty surgery itself. The remaining complications were due to other nonsurgical factors such as deep vein thrombosis, thromboembolism, and pulmonary complications. There were also other neurological complications such as dysphagia, dysrhythmia, and paralysis that may have been related to the cranioplasty surgery or underlying neurological impairment. However, this could not be determined from the study cited. Therefore, this 26% is likely an underestimation of true cranioplasty-related complications in this cohort [1]. Patients who experience cranioplasty surgery-related complications often report negative impacts on their quality of life (QOL), including pain, impairments in facial function, emotional distress, worsened work life, and appearance-related concerns [8, 9].

Further, patients undergoing cranioplasty are from a diverse population in regard to etiology, anatomy, comorbidities, and/or neurological and functional deficit. These factors may contribute to impaired QOL regardless of cranioplasty outcomes. Although these deficits may not be corrected by a cranioplasty procedure, the contribution of brain parenchymal impairment versus symptoms due to syndrome of the trephined has not been clearly elucidated in the literature. Thus, if there is a possibility that overall QOL may be improved by cranioplasty, it certainly warrants evaluation.

To comprehensively evaluate the risks and benefits of cranioplasty, it is crucial to understand patients’ perspectives through QOL assessment tools such as patient-reported outcome measures (PROMs). PROMs are standardized questionnaires that obtain information directly from patients about their symptoms and functional status, without any interpretation by a clinician or anyone else. When rigorous, validated PROMs are implemented in clinical care, they can be used to understand patient concerns and treatment preferences, guide preoperative counseling discussions, ensure goal-concordant care, conduct comparative clinical- and cost-effectiveness research, and for quality improvement initiatives, ultimately resulting in patient-centered decision-making and higher treatment satisfaction [10].

When selecting a PROM for use in patients undergoing cranioplasty, it is important to ensure that the PROM has evidence of established measurement properties (reliability, validity, and responsiveness) in the population of interest. This is crucial to evaluate outcomes and generate useful and realistic expectations to improve surgical outcomes [11]. Therefore, to provide the best care for these patients, it is critical to understand a patient’s perspectives of their QOL in a form of a validated QOL PROM to fully understand the perspective on the impacts of their specific cranioplasty procedure irrespective of their underlying condition.

Although various studies have used generic and condition-specific PROMs to evaluate QOL in patients who undergo cranioplasty, to our knowledge, there has been no systematic evaluation to assess if the properties of these PROMs are adequate to detect QOL changes pertinent to this patient population. Thus, the objectives of this systematic review were to (1) identify, critically appraise, and analyze the content of all validated PROMs for cranioplasty and (2) describe patient-reported outcomes measures utilized in adult cranioplasty patients.

## Materials and methods

### Literature search strategy

A comprehensive, electronic search of PubMed, Embase, CINAHL, and PsycINFO databases was performed last October 10, 2022. The search strategy was designed with the help of a research librarian at the Ohio State University and designed in PubMed/Medline using Medical Subject Headings (MeSH) and keywords that relate to the following four concepts: craniectomy, cranioplasty, patient-reported outcomes, and quality of life. The PubMed/Medline search strategy was later formatted according to the requirements of the additional databases. The comprehensive list of search terms can be found in “Additional Material.”

### Study inclusion criteria

Covidence, a review management program, was used to screen studies. Search results from each database were uploaded into Covidence, and duplicates were removed. Articles were included if they (1) investigated adults (≥18 years) who underwent a cranioplasty procedure, (2) used a validated PROM(s) that measured QOL, (3) conducted any assessment of the measurement properties ( e.g., content validity, construct validity, and reliability) of the PROM(s), and (4) the PROM items could be extracted from the literature. Articles were excluded if they were animal studies, case reports, literature reviews, conference proceedings, commentaries, or not available in English. All articles that used PROMs but did not satisfy all the eligibility criteria were excluded. At least two independent reviewers (TZ, NB, DR, JT, IZ, or KSM) screened title and abstracts followed by full-text review of the relevant studies. In the case of conflicting screening outcomes, a consensus was reached by discussion with an author with expertise in PROM research (MK) or the senior author (KSM).

### Data extraction and analysis

The following information was extracted from articles: author, publication year, sample size, study design, the primary objective of the study, type of cranioplasty material, the indication of cranioplasty, QOL PROMs used, and any available preoperative and postoperative QOL PROM results.

We used articles referenced by the authors of included studies, Google Search, or a snowballing approach to locate QOL PROMs used in the articles. The following information was extracted from a pilot study of the PROM or PROM website: year of original PROM publication, properties measurements, target population, number of items, and characteristics of items. Data were uploaded to Microsoft Excel where two independent reviewers (TZ, NB) categorized the items by overarching concepts, and each item was placed in four categories and eight different subdomains, physical health (physical function, pain, and energy/sleep), psychological health (psychological symptoms, memory, and bodily image/appearance), social health (social function and relationships), and general QOL.

## Results

### Literature search

**Fig. 1:**
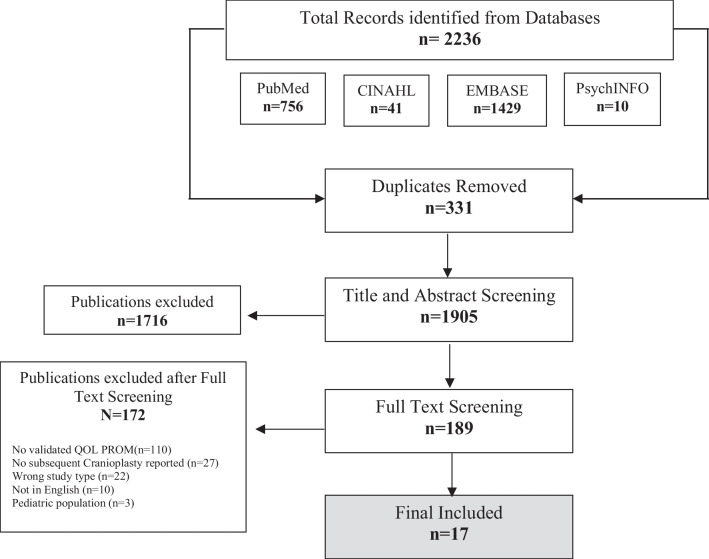
PRISMA flow diagram of search and study selection. Shows the flow of study identification and selection. The original database search resulted in 2236 articles, and 331 duplicates were automatically removed. The first phase of screening was title and abstracts screening, and 1716 articles were removed. The second phase of screening was full-text screening resulting in l 17 articles that met inclusion criteria. Abbreviations: PROM, patient-reported outcome measures; QOL, quality of life

This systematic review identified 2236 articles after the removal of duplicates (*n*=331). After the title and abstracts were screened, 1716 articles were removed, leaving 189 publications for full-text review. After the full-text screening, 17 articles and eight QOL PROMs met the inclusion criteria and were included in the systematic review. Figure 1 illustrates the Preferred Reporting Items for Systematic Review and Meta-Analysis (PRISMA) flow diagram for the literature search results. Table 1 summarizes the included articles in accordance with the population, intervention, comparison, outcome, and study (PICOS) design framework [12–28].

**Table 1:** Articles using a PROM to evaluate QOL in adults who underwent cranioplasty

Article	Study design	Population assessed	Number of patients who underwent cranioplasty	Cranioplasty material	Indication of cranioplasty	Outcome measured	PROMs used	Results
Giese, 2021 ^9^	Retrospective study	Adults treated with cranioplasty	94	Material not stated	• TBI: 24 • Infection: 13 • Ischemia: 28 • Hemorrhage: 25	Neurological and cosmetic outcomes as well as QOL	SF-36 EQ-5D	1/3 of patients had a good QOL regarding the physical and mental summary score. ½ of patients had a poor QOL in comparison to the healthy Worse results were observed in patients after ischemia CP
Foerch, 2004 ^7^	Prospective study	Space-occupying middle cerebral artery (MCA) infarction who underwent DC and subsequent CP	36	Autologous bone: 36	Ischemia: 36	Functional impairment, disability, and health-related QOL	SA-SIP 30	Overall, we found a marked reduction of QOL after ischemia
Ganau, 2020 ^8^	Retrospective study	Adults treated with cranioplasty	218	Titanium: 21 PEEK: 14 Acrylic: 2 PHA: 92 PMMA: 89	• TBI: 137 • Ischemia: 35 • Tumor: 14	Trends in CP material complications rate and surgical outcomes	SF-36	No statistically significant changes were appreciated between the different physical and mental tests except for a better psychological in the PHA CP group
Giese, 2020 ^10^	Prospective study	Adults treated with cranioplasty	67	PMMA: 67	• Implant failure: 39 • Infection: 10 • Tumor: 18	Use of PMMA in CP	SF-36 EQ-5D	86.2% of the patients showed no or only moderate impairment of mobility
Honeybul, 2013 ^15^	Prospective study	TBI, DC and subsequent CP with severe disability	20	Material not stated		QOL	SF-36	QOL mental scores of the patients were reasonably high (median 46, interquartile range 37–52)
Intisco, 2011 ^16^	Retrospective study	TBI who underwent DC with intractable cerebral edema and subsequent CP	44	Material not stated	TBI: 44	QOL	SF-36	No significant differences between the groups, the patients with TBI did report higher mean scores than those with CH in all domains
Lindner, 2017 ^19^	Randomized clinical trial	Adults treated with cranioplasty	53	Titanium: 26 HA: 26	• TBI: 5 • Implant Failure: 3 • Infection: 14 • Ischemia: 2 • Tumor: 4 • Hematoma: 10 • Aneurysm: 3 • Csf Leak: 2 • Osteonecrosis: 9	Compare local and systemic infections related to the implant and compare reoperation rate, complication rate, clinical and neurological outcomes, and health-related QOL	SF-36	58% of the patients in the titanium group felt the same or better in 1 year vs 79% in the HA group Both groups were more satisfied at the end of the study than at the beginning with a greater increase in satisfaction in the titanium implant group
Low, 2019 ^20^	Prospective study	Adults treated with cranioplasty	11	PMMA: 11	TBI: 9 Hemorrhage: 2	Develop a new technique for cranioplasty candidates utilizing computer-assisted 3D modeling and printing	SF-36	Mean SF-36 score improved following surgery There was a positive correlation between the cosmetic result (VASC) and improvement in role limitations due to emotional problems
Malmivaara, 2011 ^21^	Retrospective study	TBI treated with DC with subsequent CP	30	Material not stated	TBI; 30	Cost-effectiveness	EQ-5D	EQ-5D index were good, and therefore, the treatment turned out to be cost-effective
Malmivaara, 2011 (non-traumatic) ^22^	Retrospective study	Non-traumatic DC and subsequent CP	20	Autologous bone: 20	Unknown	Costs of all emergencies DC	EQ-5D	Patients has mean lower HRQOL compared to the general population
McKenna, 2012 ^24^	Case series	Malignant middle cerebral artery infarction treated with DC and subsequent CP	6	Autologous bone: 6	Ischemia: 6	Neuropsychological and psychosocial outcomes	Stroke and Aphasia Quality of Life-39	Normative data based on 83 individuals with aphasia (resulting from a stroke) of at least 1 year duration who had no known premorbid history of severe cognitive decline or mental health problems and who were living at home before the stroke
Schmidt, 2011 ^31^	Retrospective study	Complete media infarction of the non-speech-dominant hemisphere treated with DC and subsequent CP.	20	Autologous bone: 20	Ischemia: 20	Neuropsychological deficits, QOL and the extent of depression and other psychiatric symptoms	WHOQoL-Bref	Mean *z* values for the WHOQoL questionnaire displayed a clinically significant impairment of the health-related QoL
Sundseth, 2015 ^34^	Retrospective study	Speech-dominant and non-dominant side infarction. Treated with DC and subsequent CP	24	Autologous bone: 24	Ischemia: 30	This study compared functional outcome, QOL and mental health	SF-36	Patients performed comparable to the general US population scores from 1998
Ungar, 2020 ^36^	Case series	Adults treated with cranioplasty for anterior skull base deficit	12	Soft tissue free flap: 12	• Infection: 1 • Tumor: 10	Present a method of median anterior skull base (ASB) reconstruction using a sub cranial approach with a free flap	Anterior Skull Base Quality-of-Life Questionnaire	Median ASB responses were comparable to those reported for patients who underwent sub cranial ASB resection without free flap
Waquas, 2018 ^37^	Observational study	TBI treated with DC and subsequent CP	34	Material not stated	TBI: 34	QOL	QOLIBRI	77% of patients were satisfied with their QOL and would give consent for the procedure again
Worm, 2019 ^38^	Prospective clinical trial	Large cranial bone defects before and after late cranioplasty	62	PMMA: 62	• TBI: 50 • Ischemia: 6 • Unknown: 4	QOL	SF-36	70% of patients returned to labor activities, regaining economic independence
Zegers, 2017 ^39^	Retrospective study	Large skull bone defects repair with titanium or PEEK	29	Titanium: 12 PEEK: 21	• TBI: 7 • Infection: 4 • Ischemia: 8 • Tumor: 7 • Epilepsy: 3	QOL, pain, esthetics, and the surgical and medical outcomes	Glasgow Benefit Inventory (GBI)	Statistically significant results could be found with regard to the general health and physical health subscale. Benefit was also seen in the social support subscale; however, without statistical significance
Totals	• Retrospective studies: 8 • Prospective studies : 4 • Case series :2 • Randomized clinical trial: 1 • Prospective clinical trial: 1 • Observational study: 1	• Cranioplasty: 9 • TBI and DC: 5 • Ischemia: 4	Total patients: 779	• PMMA: 231 • Unknown: 222 • Autologous Bone: 106 • PHA: 92 • Titanium: 55 • PEEK: 35 • Hydroxyapatite: 26 • Soft tissue free flap: 12 • Acrylic: 2	• TBI: 340 • Ischemia: 165 • Tumor: 55 • Implant failure: 42 • Cerebral hemorrhage: 27 • Unknown:24 • Infection: 13 • Hematoma: 11 • Aneurysm: 3 • Epilepsy: 3 • Csf leak: 2 • Osteonecrosis: 9		• SF-36: 9 • EQ-5D: 4 • Other PROMS appeared only 1 time	

Summarizes the seventeen articles that met inclusion criteria according to the population, intervention, comparison, outcome, and study (PICOS) design

Abbreviations: *TBI* traumatic brain injury, *QOL* quality of life, *CP* cranioplasty, *PEEK* polyether ether ketone, *PHA* polyhydroxy alkanoates, *PMMA* polymethyl methacrylate, *DC* decompressive craniectomy, *CSF* cerebral spinal fluid, *ABS* anterior skull base, *HRQOL* health-related quality of life

### Analysis of articles retrieved

In the 17 articles that met inclusion criteria, there were a total of 779 patients who underwent cranioplasty with subsequent evaluation of QOL in the form of a PROM. The primary aim of ten of the 17 studies was to investigate aspects of patients QOL, whereas the remaining seven studies primarily aimed to investigate other outcomes such as the cost of the procedure and outcomes of cranioplasty material, with the secondary aim of evaluating QOL. In studies where the primary aim was evaluating QOL, pre- and post-QOL PROM results showed improvements in QOL after cranioplasty (Table 1).

The major indications for cranioplasty were to repair cranial defect following decompressive craniectomy (DC) for traumatic brain injury (TBI) (*n*=340) and cerebral ischemia (*n*=165). The most common type of cranioplasty material used among the 779 patients was a poly(methyl methacrylate) (PMMA) implant (*n*=239), followed by autologous bone (*n*=106) (Table 1).

The population of interest in eight of the 17 articles was patients who underwent cranioplasty to repair cranial defect. For the remaining articles, the population of interest was patients who had TBI (*n*=9), TBI, and DC (*n*=5) or cerebral infarction and DC (*n*=4), with a secondary aim of evaluating patients after subsequent cranioplasty (Table 1). The number of previous cranial operation(s) was not consistently reported and therefore could not be extracted.

Finally, all 17 articles used existing PROMs to evaluate QOL, and none of the articles used newly developed QOL PROMs or assessed psychometric principles of the existing PROMs to ensure the PROMs had content validity, reliability, construct validity, or responsiveness in those who underwent cranioplasty.

### PROM psychometric assessment

Although all eight QOL PROMs demonstrated validation in the original pilot study as well as various other condition-specific studies, we did not find any evidence of content validity, construct validity, or criterion validity for use in adults with cranioplasty in the original or condition-specific studies. Table 2 summarizes the eight QOL PROMs extracted from the 17 articles [29–35]. Three of the eight QOL PROMs retrieved were generic, and the other five were developed for a specific condition (Table 2). The number of items used in each questionnaire ranged from 5 to 38. The most utilized questionnaires among the 17 articles were Short Form Health survey 36 (SF-36) (*n*=9), which is a widely used generic QOL PROM.

**Table 2:** QOL PROMs used in patients who underwent cranioplasty

Questionnaires	Country of development	Year of validation	Target population	Item number	Validation demonstrated	Measurement domains	Publication or website
EuroQol- 5 Dimension (EQ-5D)	Multi-country	1990	Generic	5	Concurrent validity, convergent validity, known-group validity, and responsiveness	Mobility (1 item) Self-care (1 item) Usual activities (1 item) Pain/discomfort (1 item) Anxiety/depression (1 item)	https://euroqol.org/eq-5d-instruments/
Glasgow Benefit Inventory (GBI)	Scotland	1996	Otolaryngology	18	Construct Validity and reliability	General health (12 items) Social support (3 items) Physical health (3 items)	Robinson ^30^
Quality of Life After Traumatic Brain Injury (QOLIBRI)	Multi-country	2010	TBI	37	Construct validity, internal consistency, and test–retest reliability	Cognition (7 items) Self (7 items) ADLs and autonomy (7 items) Social relationships (6 items) Emotions (5 items) Physical problems (5 items)	Von steinbuchel ^32^
Short Form Health Survey (SF-36).	UK	1992	Generic	36	Internal consistency, test–retest reliability, convergent, and discriminant validity	Physical functioning (10 items) Physical role limitations (4 items) Bodily pain (2 items) General health perceptions (5 items) Energy/vitality (4 items) Social functioning (2 items) Emotional role limitations (3 items) Mental health (5 items) Perceived changed in health (1 item)	Brazier ^5^
Stroke & Aphasia Quality of Life-39 Scale	UK	2002	Patients with aphasia	39	Construct validity, internal consistency, and test–retest reliability	Physical (17 items) Psychosocial (11 items) Communication (7 items) Energy (4 items)	Hilari ^13^
WHOQoL-Bref	Multi-country	1998	Generic	26	Discriminant validity, content validity, and test–retest reliability.	Physical health (3 items) Psychological (5 items) Levels of independence (4 items) Social relationships (3 items) Environmental (8 items)	WHOQOL^40^
Anterior Skull Base Quality-of-Life Questionnaire	Multi-country	2004	Patients with anterior skull base injury	35	Internal consistency, test–retest validity	Role of performance (6 items) Physical function (7 items) Vitality (7 items) pain (3 items) Specific symptoms (7 items) Impact on emotions (5 items)	Gil ^11^
Stroke-adapted short form (the SA-SIP 30)	Netherlands	1997	Stroke	30	Construct validity, convergent validity, external validity	Body care and movement (5 items) Social interaction (5 items) Mobility (3 items) Communication (3 items) Emotional behavior (4 items) Household management (4 items) Alertness behavior (3 items) Ambulation (3 items)	Van Straten ^33^

Summarizes the eight QOL PROMs that met inclusion criteria, country of development, year of validation, target population, item number, validation demonstrated, and measurement domain with item number

Abbreviations: *ADLs* activities of daily living

### Content analysis of PROMs

**Fig. 2:**
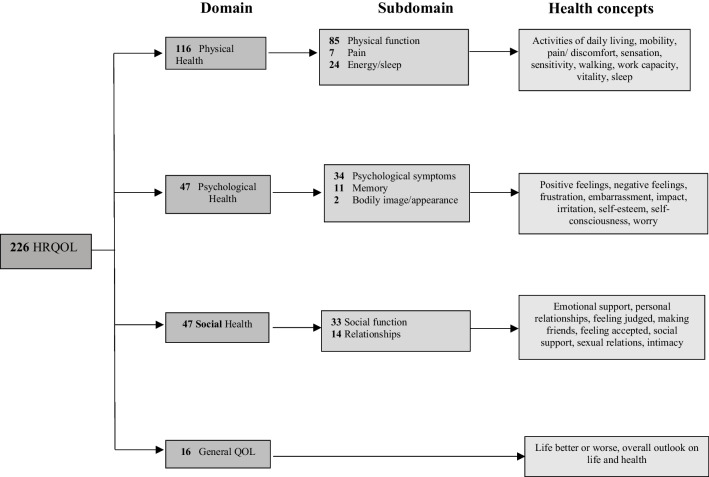
PROM items used to assess patients who underwent cranioplasty. Is a conceptual framework-based on the content of the items found in eight of the QOL PROMs retrieved. Abbreviations: HRQOL, health-related quality of life; QOL, quality of life; PROMs, patient-reported outcome measures)

A total of 226 items from the eight QOL PROMs were identified. Figure 2 shows a literature-informed conceptual framework for the 4 domains of physical health (116 items), psychological health (47 items), social health (47 items), and general QOL (16 items) measured in the included studies.

Physical health was measured by 116 items. Of these, 85 items aimed to evaluate physical function and mobility such as “I have slight problems in walking about”; seven items involved pain such as “How much bodily pain have you had during the past 4 weeks?”; and 24 items aimed to measure energy/sleep such as “Feeling tired or having little energy.” Psychological health was measured by 47 items among the eight PROMs. Of these, 34 aimed to evaluate psychological symptoms such as emotional distress, for example, “Have you felt so down in the dumps that nothing could cheer you up?” Eleven items were geared towards assessing memory such as “Do you have difficulties with thinking and memory?” Satisfaction with appearance was measured by two items in only two of eight PROMS (WHOQoL-Bref and the Anterior Skull Base Quality-of-Life Questionnaire) (Table 2) with questions such as “How do you feel about your bodily image/appearance?” Social health was measured by 47 items among the eight different PROMs. Of these, 33 items evaluated general social function such as “Since your operation, have you been more or less inclined to withdraw from social situations?”, and 14 items assessed relationships such as “How satisfied are you with your personal relationships?” General QOL was measured by 16 items among the eight different PROMs evaluating how patients view their overall life and health with questions such as “How would you rate your quality of life?”

## Discussion

To our knowledge, this is the first systematic literature review to determine the content of validated PROMs used to evaluate QOL in adult patients undergoing cranioplasty. Through this review, we identified eight PROMs that have been used to evaluate QOL in patients who underwent cranioplasty. None of the PROMs identified was specifically developed or validated for use in this specific patient population. This lack of validated PROMs used to evaluate QOL in patients who undergo cranioplasty may be hindering consensus regarding the best approach to provide optimal care for these patients.

Findings from our content analysis were used to provide a literature-informed conceptual framework for patient-reported outcomes. However, despite the known importance of undergoing cranioplasty to restore the form and function of the skull, these items fail to measure facial function (such as facial expression or nonverbal communication) and only two of the 226 items measured appearance. Our review identified three generic PROMs and five condition-specific PROMs. While generic PROMs are beneficial, they may exclude specific concerns of the patient population being evaluated. Furthermore, the items in the PROMs were lacking items tailored to patients’ surgical experience, such as the occurrence and severity of adverse events. This makes it difficult to determine patients’ satisfaction with their specific cranioplasty surgery. These concepts encompass key aspects of content validity, that is, the degree of relevance, comprehensiveness, and comprehensibility of an instrument in the context of a target population. The content validity is considered the most important measurement property of a PROM because if the items in the PROM do not capture concepts that are meaningful to the patient, it does not matter if the PROM is reliable or has construct validity [35].

Finally, of the articles retrieved, only three studies meeting inclusion criteria were primarily aimed to investigate QOL in adult patients undergoing cranioplasty. This paucity of studies utilizing PROMS to specifically investigate QOL shifts the focus on surgeon-reported outcomes such as complications (for example, reoperation(s) to correct temporal hollowing deformity). However, with the increasingly important role of PROMs in clinical practice, it is necessary to capture patient experience and perspective on the quality and impact of their treatment and care. Using PROMs can provide insights on patient experience to guide clinical practice while also serving as a paramount tool to engage in discussions with regulatory agencies, lawmakers, and payers and support an evidence-based approach to treatment [36].

Cranioplasty studies focused on surgical outcomes such as rates of infection and functional status are extremely valuable outcomes to study and understand. However, other variables that are important to patients must also be considered. For instance, in cranioplasty, a patient may have a durable and sustainable cranial implant with low rates of complication, but they may have facial function deficiency or may be insecure about their skull contour, scars, and alopecia [37]. Thus, gathering patient-reported outcomes such as QOL after cranioplasty is necessary to assess and create an individualized and patient-centered care plan.[38] Additionally, with bioengineering and technology becoming increasingly important to optimize care in cranioplasty, QOL PROMS can be used as an endpoint in clinical trials as the Food and Drug Administration (FDA) allows treatments to be approved based on QOL data in addition to survival or adverse event outcomes. [36]

Finally, in this review, we aimed to focus on QOL measures because there is a paucity of studies as well as validated tools assessing patients undergoing cranioplasty. Certainly, with cranioplasty being performed for a variety of reasons, one may ask whether different PROMs are needed for different etiologies. However, regardless of the indication for cranioplasty, a major component of the procedure is to restore patients to a lifestyle where they do not have to wear helmets, they can participate in physical activities, they may return to work, and overall they have improved social performance, all of which are critical components of QOL regardless of the etiology of their underlying condition. This call to action to create a specific cranioplasty tool will increase patient exposure to more questionnaires and testing and to the health care system. However, a validated PROM that broadly assesses cranioplasty outcomes may be utilized in combination with a more condition-specific questionnaire to mitigate survey fatigue.

One of the main limitations of this study is the small number of studies with the primary aim of assessing QOL in cranioplasty. It was not possible to conduct a meta-analysis or compare outcomes in the included studies due to three main reasons. Firstly, there was substantial variability in the methodological approach and reporting of patient cohort and outcomes. Secondly, at a conceptual level, all the PROMs varied in their definition of what constitutes QOL. Lastly, there was heterogeneity in the different management protocols and materials used to reconstruct the skull. Despite these limitations, the results of this systematic review reflect the need for a rigorously developed validated QOL PROMs specific to this patient population.

## Conclusion

Cranioplasty is a life-enhancing and potentially lifesaving procedure that aims to positively impacts patients’ QOL. Given the high rate of complications, a multidisciplinary team consisting of neurosurgeons, plastic surgeons, craniofacial surgeons, and/or neuroplastic surgeons is often required.

Utilizing validated PROMs to assess QOL can ensure cohesiveness throughout patient management. Further, a standardized QOL measurement is useful for patient–clinician discussions to help set realistic expectations and inform clinical decision-making to improve patient outcomes [39]. In this review, we found there is a paucity of condition-specific validated PROMs for use in cranioplasty. This reflects the need for the development of items and concepts that can truly target concepts important to these patients. This review is a call to action and will serve as a foundation for future development of a valid, reliable, and condition-specific PROM for patients undergoing cranioplasty.
